# Honokiol Provides Cardioprotection from Myocardial Ischemia/Reperfusion Injury (MI/RI) by Inhibiting Mitochondrial Apoptosis via the PI3K/AKT Signaling Pathway

**DOI:** 10.1155/2022/1001692

**Published:** 2022-03-27

**Authors:** Linhua Lv, Qiuhuan Kong, Zhiying Li, Ying Zhang, Bijiao Chen, Lihua Lv, Yubi Zhang

**Affiliations:** ^1^Department of Cardiac Surgery, The First Affiliated Hospital of Sun Yat-sen University, Guangzhou, 510080 GD, China; ^2^Department of Internal Medicine, Sun Yat-sen University Cancer Center, Guangzhou, 510080 GD, China; ^3^Department of Pediatrics, The First Affiliated Hospital of Sun Yat-sen University, Guangzhou, 510080 GD, China; ^4^Department of Pharmacy, Shaoyang University, Shaoyang, 422000 HN, China; ^5^Department of Hematology Oncology, Shaoyang Central Hospital, Shaoyang, 422000 HN, China

## Abstract

**Background:**

Myocardial injury refers to a major complication that occurs in myocardial ischemia/reperfusion injury (MI/RI). Honokiol is a well-recognized active compound extracted from the traditional Chinese herb known as *Magnolia officinalis* and is utilized in treating different vascular diseases. This research is aimed at examining whether Honokiol might alleviate myocardial injury in an MI/RI model.

**Methods:**

Seventy-eight male C57BL/6 mice were categorized randomly into three cohorts including the Sham operation (Sham) cohort, the MI/RI cohort (Con), and the Honokiol cohort (*n* = 26 for each cohort). The mice in the Honokiol cohort were treated with Honokiol before MI/RI surgery (0.2 mg/kg/day for 14 days, intraperitoneal), while the mice in the Con cohort were given an intraperitoneal injection with an equivalent volume of vehicle (DMSO) daily in 14 days prior to exposure to MI/RI. After the surgery, creatine kinase- (CK-) MB and cardiac troponin T (cTnT) levels, as well as the infarct area, were measured to assess the degree of myocardial damage. Apoptotic levels were detected using terminal deoxynucleotidyl transferase dUTP nick-end labeling (TUNEL) staining. Electron microscopy was utilized to identify mitochondrial damage. Lastly, the expression levels of glyceraldehyde-3-phosphate dehydrogenase (GAPDH), cleaved caspase-9, cytochrome C (Cyt-C), B cell lymphoma/leukemia-2 (Bcl-2), B cell lymphoma/leukemia-2 associated X (Bax), AKT, p-AKT, PI3K, and p-PI3K were analyzed utilizing western blotting.

**Results:**

Honokiol can reduce the MI/RI-induced cTnT and CK-MB levels, apoptosis index, and mitochondrial swelling in cardiomyocytes via activating the PI3K/AKT signaling pathway.

**Conclusion:**

Honokiol provides cardiac protection from MI/RI by suppressing mitochondrial apoptosis through the PI3K/AKT signaling pathway.

## 1. Introduction

Myocardial ischemia/reperfusion injury (MI/RI) is a high degree of organ injury that may result from restoring blood flow following cross-clamping in cardiopulmonary bypass (CPB) in the process of heart surgery and after revascularization therapy postmyocardial infarction (MI) [1]. MI/RI promotes the development of reactive oxygen species (ROS) and calcium overload, both of which may lead to the production of cytochrome C (Cyt-C) into the cytoplasm [2]. This is followed by the activation of caspases, subsequently resulting in mitochondrial dysfunction and swelling and, eventually, apoptosis [3–5]. Hence, discovering effective methods of pharmacological preconditioning to reduce mitochondrial damage and cell apoptosis may be an effective therapy to alleviate MI/RI [6–8].

Honokiol has been known as an active compound extracted from the traditional Chinese herb commonly referred to as *Magnolia officinalis*, which is utilized in the treatment of different vascular diseases including heart disease, stroke, and ischemia [9–11]. Early investigations showed that Honokiol limits infarct size and has antiarrhythmic impacts in rats with acute MI [12, 13]. Additionally, Honokiol was found to perform a function in enhancing postischemic cardiac functions, lessening infarct size, lowering myocardial apoptosis, and shrinking ROS levels in MI/RI injury in type 1 diabetes [14]. In addition to this, pretreatment using Honokiol also substantially decreased the infarct size, as well as the levels of serum creatine kinase (CK), nuclear factor *κ*B (NF-*κ*B), interleukin- (IL-) 6, tumor necrosis factor- (TNF-) *α*, and lactate dehydrogenase (LDH), in an MI/RI rat model [15]. Currently, studies about the mechanisms of the cardioprotective effects of Honokiol on MI/RI mainly point to inflammation as well as oxidative stress.

In this research, we examined the function of Honokiol in preventing MI/RI, particularly its potential mechanism of decreasing mitochondrial damage in cardiomyocytes.

## 2. Methods

### 2.1. Mouse Care

Adult male C57BL/6 mice (aged between 8 and 12 weeks and weight ranging between 20 and 25 g) were procured from GemPharmatech Co. Ltd. The mice were kept under a steady temperature of 22 ± 2°C and humidity (45 ± 5%), with a cycle of 12-hour daylight and 12-hour darkness, and an unrestricted supply of water and food. All the experimentations were carried out as per the Guide for the Care and Use of Laboratory Animals by the National Academy of Sciences, published by the National Institutes of Health (NIH Publication No. 86-23, revised 1996), and certified for use by the Institutional Animal Care Committee of Shaoyang University.

### 2.2. Mouse Treatment and Surgical Procedure

Adult male C57BL/6 mice used in the experiment were categorized randomly into three cohorts, which include the Sham operation cohort (Sham, *n* = 26), the MIRI cohort (Con, *n* = 26), and the Honokiol cohort (Honokiol, *n* = 26).

Animals in the Honokiol cohort were treated with Honokiol before MI/RI surgery (0.2 mg/kg/day for 14 days, i.p.), whereas the mice in the Con cohort were given an intraperitoneal injection with an equivalent volume of vehicle (dimethyl sulphoxide (DMSO)) each day for 14 days prior to the exposure to MI/RI. The dosage of Honokiol used in this research was based on previous study [16]. An equivalent volume of DMSO was given to the Con cohort. After anesthetizing with 2% Nembutal sodium (50 mg/kg), the mice underwent artificial ventilation via endotracheal intubation (110 breaths/min, 0.2 ml tidal volume) using a rodent ventilator. Then, the left thoracotomy was carried out in the 4^th^ intercostal space, and the left anterior descending (LAD) coronary artery was occluded by a 10-0 PROLENE® suture for half an hour ensued by 2 hours of reperfusion after removal. The mice in the Sham cohort were subjected to a similar operation, with the exception of ligation of the coronary artery. After reperfusion, the mice were sacrificed via exsanguination after treatment with 2% Nembutal sodium. After blood extraction, the plasma was isolated through centrifugation, and after isolation, the plasma was kept under a temperature of −80°C before being used. Mice hearts were harvested after euthanasia for further evaluation.

### 2.3. Measurement of cTnT and CK-MB Levels

The plasma levels of CK-MB and cTnT were estimated and analyzed to assess the degree of myocardial damage. The cTnT levels were evaluated utilizing a high-sensitivity mouse cTnT enzyme-linked immunosorbent assay (ELISA) kit (Life Diagnostics, West Chester, PA, USA) as per the instructions stipulated by the manufacturer. On the other hand, CK-MB levels were estimated utilizing an automated analyzer (Chemray 800, Rayto Life and Analytical Sciences, Shenzhen, China).

### 2.4. Estimation of the Myocardial Infarct Area

After the completion of the reperfusion procedure, the myocardial infarct size was examined utilizing a double-staining technique called 2,3,5-triphenyl tetrazolium chloride- (TTC-) Evans blue staining. In this method, sections stained with blue signifies the nonischemic section, the red staining signifies the risk section, and the white sections represent the infarct section. After reperfusion, the coronary artery was again occluded using the PROLENE® suture, and 1 ml of 2 percent Evans blue solution (Sigma) was reversely introduced into the aorta. The hearts were quickly removed, kept at a temperature of −20°C for 20 min, and subsequently sliced into slices of 1 mm. The heart sections were subjected to incubation using a solution of 1% TTC (Sigma) for 20 min. After staining, a stopping solution (ice-cold sterile saline) was added, followed by fixing the slices in 10% neutral-buffered formaldehyde. An evaluator blinded to the identity of the specimen captured the images of both sides of each slice and then analyzed the images utilizing the Image-Pro Plus 6.0 software (Media Cybernetics, Silver Spring, MD, USA).

### 2.5. Echocardiography

Assessment of the heart function was done at the end of the 2-hour reperfusion utilizing transthoracic echocardiography (VisualSonics system, Toronto, Ontario, Canada). In this procedure, cardiac variables, such as cardiac output, ejection fraction, LV fractional shortening, wall thickness, and left ventricular (LV) end-diastolic dimension, were assessed via M-mode and two-dimensional echocardiography. Anesthetization of the mice was done using 1.5% isoflurane/oxygen prior to the procedure.

### 2.6. TUNEL Staining of Apoptotic Cells

Fixing of the hearts was done in 4-percent paraformaldehyde, entrenched in paraffin, followed by sectioning at a width of 5 *μ*m. Subsequently, *in situ* apoptosis was evaluated through the TUNEL technique utilizing an *In Situ* Cell Death Detection Kit, Fluorescein (Roche Applied Science, Mannheim, Germany). Succinctly, the slices were subjected to washing 3 times with 10 mM phosphate-buffered solution (PBS) ensued by permeabilization in proteinase K for a duration of 10 minutes. After washing another 3 times, incubation of the sections was done in TdT buffer at the temperature of 37°C for 1 hour and then with antibodies using the same parameters. Afterward, staining of the cell nuclei was done with 4,6-diamino-2-phenylindole (DAPI). The apoptotic cardiomyocytes are represented by TUNEL/DAPI-positive cells. Sections were randomly selected, and five areas were randomly selected from each section to estimate the percentage of apoptotic cells. The percentage of TUNEL-stained nuclei was computed as the proportion of the amount of TUNEL-positive nuclei/total nuclei. Quantification of the aggregate amount of apoptotic nuclei was done through tallying the overall number of TUNEL-positive nuclei in whole slices from seven distinct mice hearts per cohort. The photographs were taken utilizing a Zeiss digital camera attached to a Zeiss VivaTome microscope (Zeiss, Thornwood, NY, USA). The section images were then analyzed utilizing the ImagePro Plus 6.0 software (Media Cybernetics).

### 2.7. Electron Microscopy

The specimens were submerged in 2.5 percent glutaraldehyde followed by postfixing in 2 percent osmium tetroxide in sodium phosphate buffer for a duration of 2 hours at a temperature of 4°C. Next, dehydration of the specimens was done in a graded sequence of ethanol and propylene oxide and entrenched in araldite. An ultramicrotome (Leica EM UC7; Leica, Nussloch, Germany) was utilized to cut 1 *μ*m sections. The sections were subjected to staining utilizing uranyl acetate and lead citrate and then viewed utilizing a Hitachi transmission electron microscope (HT7700; Hitachi, Tokyo, Japan).

### 2.8. Western Blotting (WB)

The protocol was previously described [17–19]. A lysis buffer (Beyotime, Shanghai, China) containing a protease inhibitor cocktail (Millipore, Billerica, Massachusetts, USA) was utilized to isolate proteins from cardiac tissues. Subsequently, the proteins were analyzed utilizing sodium dodecyl sulfate-polyacrylamide gel electrophoresis (SDS-PAGE) in accordance with the basic instructions and then moved to polyvinylidene difluoride (PVDF) membranes to perform WB analysis (Millipore). Primary antibodies against Bcl-2 (Cell Signaling Technology (CST), Danvers, MA, USA), Bax (CST), Cyt-C (CST), caspase-9 (Abcam, Cambridge, MA, USA), PI3K (CST), p-PI3K (CST), AKT (CST), and p-AKT (CST) were used for protein detection. Later, incubation of the membranes was done using an HRP-conjugated secondary antibody (Thermo Fisher Scientific, Waltham, MA, USA) for 1 hour at ambient temperature, and detection of antigen-antibody complexes was done utilizing a WB luminol reagent (Sigma-Aldrich). For this experiment, glyceraldehyde-3-phosphate dehydrogenase (GAPDH) (Proteintech, Rosemont, IL, USA) acted as an internal reference. The mean light density for each band was analyzed utilizing the ImageJ software. The target genes expression was standardized to the expression of GAPDH.

### 2.9. Statistical Analysis

Statistical analysis was carried out utilizing SPSS v. 23.0 (IBM Corp, Armonk, NY, USA). The Student *t*-test was carried out to evaluate the variations between the treatment and the sham cohorts. Comparisons among the three cohorts were done utilizing one-way ANOVA ensued by Tukey's post hoc test. Data are articulated as mean values ± standard error of the mean (SEM). For all the tests, a *p* value of <0.05 was deemed statistically significant.

## 3. Results

### 3.1. Honokiol Pretreatment Preserves Cardiac Function in MI/RI

**Figure 1:**
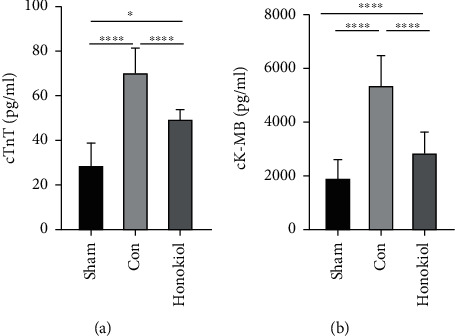
Plasma levels of myocardial injury markers. Expression levels of (a) cTnT and (b) CK-MB. Sham: sham operation; Con: MI/RI with the vehicle; Honokiol: MI/RI with Honokiol. *N* = 10 for each cohort. Data are articulated as mean ± SEM. ∗*p* < 0.05; ∗∗∗∗*p* < 0.0001.

The plasma levels of CK-MB and cTnT were considerably elevated in the Con cohort as opposed to the Sham cohort (both *p* < 0.0001, Figure 1). On the other hand, CK-MB and cTnT levels were found to be reduced in the Honokiol-treated cohort (Honokiol cohort) as opposed to the Con cohort (both *p* < 0.0001, Figure 1).

**Figure 2:**
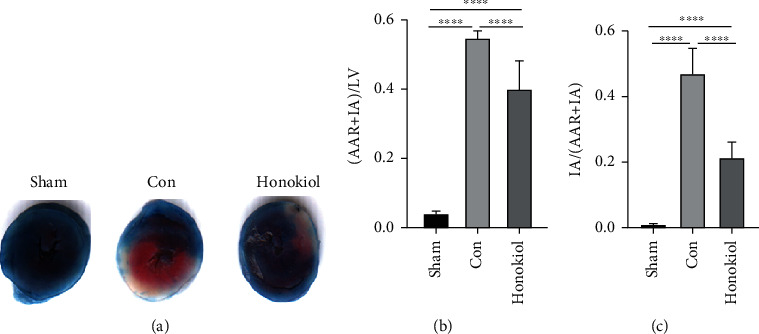
Measurement of the myocardial infarct area. (a) TTC-Evans blue staining. (b) Assessment of myocardial infarct area. Both the proportion of aggregate ischemic areas and necrotic areas were elevated in the Con cohort as opposed to the Sham cohort and were considerably decreased by Honokiol treatment. *N* = 10 for each cohort. Data are articulated as mean ± SEM. ∗∗∗∗*p* < 0.0001.

Aside from measuring the plasma cTnT and CK-MB levels, the myocardial infarct size was also estimated through TTC-Evans blue staining. The myocardial infarction severity (Figure 2(a)) was evaluated via the estimation of the proportion of the aggregate ischemic area and the proportion of necrotic areas premised on the algorithm below:

(1) $$\begin{array}{c} \%\;\text{total ischemic areas}=\frac{\left(\text{infarct plus at}‐\text{risk areas}\right)}{\left(\text{total myocardial areas}\right)}, \end{array}$$

(2) $$\begin{array}{c} \%\;\text{necrotic areas}=\frac{\left(\text{infract areas}\right)}{\left(\text{infract plus at}‐\text{risk areas}\right)}. \end{array}$$

Both of the %total ischemic areas and %necrotic areas were increased in the Con cohort as opposed to the Sham cohort but were considerably reduced in the Honokiol cohort (all *p* < 0.0001, Figure 2(b)).

**Figure 3:**
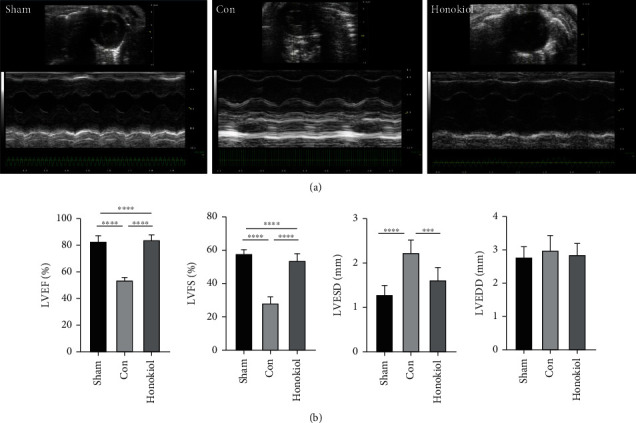
Echocardiography of MI/RI mice. (a) Echocardiograms of the mice. (b) Both LVFS and LVEF were elevated, while LVESD was lower in the Honokiol cohort as opposed to the Con cohort. No difference was observed in the LVEDD in all cohorts. *N* = 10 for each cohort. Data are articulated as mean ± SEM. ∗∗∗*p* < 0.001. ∗∗∗∗*p* < 0.0001.

Mice subjected to MI/RI (Con cohort) indicated significantly worse cardiac function as opposed to the Sham cohort, assessed by measuring the left ventricular ejection fraction (LVEF), fractional shortening (LVFS), and left ventricular end-systolic diameter (LVESD) (all *p* < 0.0001, Figures 3(a) and 3(b)). The results illustrated that both LVFS and LVEF were elevated, but LVESD was reduced in the Honokiol cohort as opposed to the Con cohort (all *p* < 0.0001, Figures 3(a) and 3(b)). No difference was identified in the left ventricular end-diastolic diameter (LVEDD) among all cohorts (Figure 3(b)).

### 3.2. Honokiol Pretreatment Reduces Cardiomyocyte Apoptosis Caused by MI/RI

**Figure 4:**
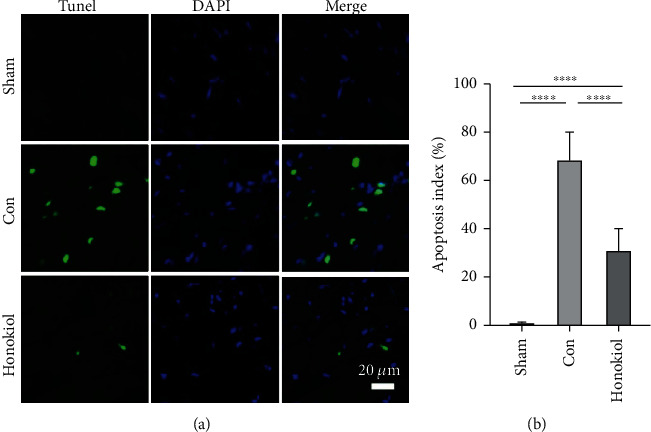
Assessment of cardiomyocyte apoptosis. The TUNEL assay was utilized to evaluate cardiomyocyte apoptosis in ventricular tissue (400x, *n* = 7 per cohort). Data are articulated as mean ± SEM. ∗∗∗∗*p* < 0.0001.

The influence of Honokiol on MI/RI-triggered apoptosis was examined utilizing TUNEL staining. As illustrated in Figure 4, only a small amount of apoptotic cardiomyocytes were identified in myocardial tissues from the Sham cohort, but a considerably higher amount of TUNEL-positive cardiomyocytes were identified in the Con cohort (*p* < 0.0001). The pretreatment with Honokiol reduced the amount of TUNEL-positive cells (*p* < 0.0001), indicating its antiapoptotic effect on MI/RI (Figure 4).

**Figure 5:**
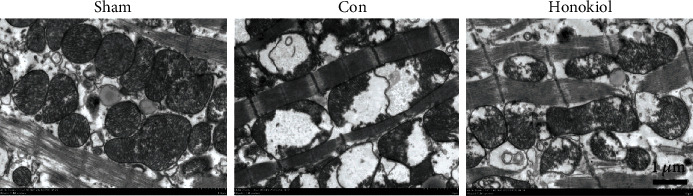
Analysis of cardiomyocytes via electron microscopy. MI/RI resulted in the damage of the myocardial ultrastructure and mitochondrial inflammation, which was attenuated after melatonin treatment (*n* = 3 for each cohort). Black arrow: mitochondria. Original magnification: 15000x.

The electron microscopy of cardiomyocytes indicated severe mitochondrial inflammation in the Con cohort but not in the Honokiol cohort (Figure 5), implying that the mitochondrial apoptosis pathway may be implicated in MI/RI.

**Figure 6:**
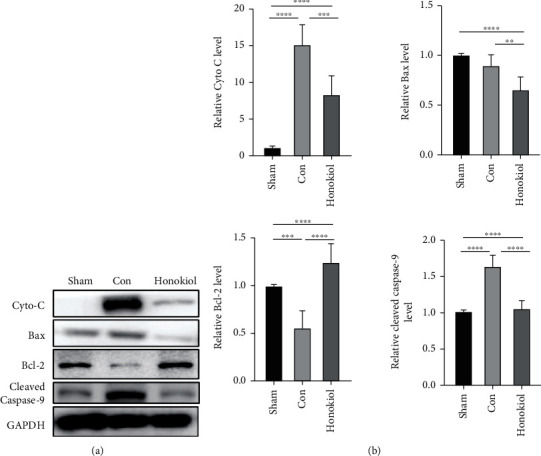
Western blot analysis of apoptosis-associated proteins. Western blotting was utilized to evaluate the protein levels of cleaved caspase-9, Cyt-C, Bcl-2, and Bax. GAPDH acted as the internal reference (*n* = 6 for each cohort). Data are articulated as mean ± SEM. ∗∗*p* < 0.01; ∗∗∗*p* < 0.001; ∗∗∗∗*p* < 0.0001.

In addition to this, mitochondrial apoptosis markers were also detected and identified through western blotting. Honokiol considerably lowered the levels of the proapoptotic proteins cleaved caspase-9, Bax, and Cyt-C, all of which are stimulated by MI/RI, but elevated the antiapoptotic protein Bcl-2 expression (all *p* < 0.05, Figure 6). These data propose that Honokiol could act as a prospective antiapoptotic drug by modulating the mitochondrial pathway for MI/RI.

### 3.3. Honokiol Activates the PI3K/AKT Signaling Pathway in MI/RI

**Figure 7:**
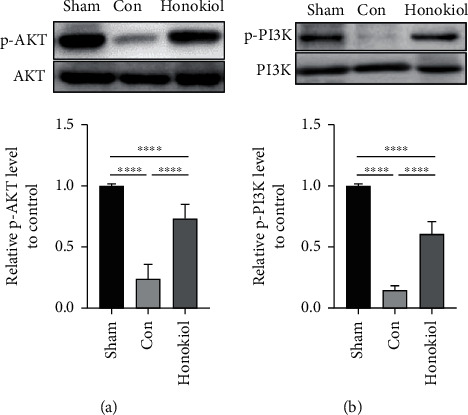
Western blot analysis of PI3K/AKT pathway-related proteins. (a) AKT and (b) PI3K were identified through WB. Sham: sham operation; Con: MI/RI with vehicle; Honokiol: MI/RI with Honokiol. *N* = 6 for each cohort. Data are articulated as mean ± SEM. ∗∗∗∗*p* < 0.0001.

The PI3K/AKT pathway can modulate cell apoptosis induced by the mitochondrial pathway [20–22]. Western blotting was performed to investigate whether Honokiol has a function in PI3K/AKT-induced mitochondrial apoptosis. It was found that the expression of p-AKT and p-PI3K in the Con cohort was significantly decreased and that Honokiol was able to restore the expression levels of the two proteins (Figure 7, all *p* < 0.0001). These results suggest that Honokiol may be preventing mitochondrial apoptosis in the cardiomyocytes through the PI3K/AKT signaling pathway.

## 4. Discussion

In this research, we have demonstrated that Honokiol pretreatment can improve cardiac function during MI/RI injury. Honokiol considerably lowered the levels of myocardial injury indicators (cTnT and CK-MB) induced by MI/RI. Furthermore, Honokiol also inhibited the cardiomyocyte apoptosis and mitochondrial swelling that resulted from MI/RI, possibly involving the PI3K/AKT signaling pathway. With everything considered, these results indicate that pretreatment with Honokiol may be protecting the heart from MI/RI by inhibiting mitochondrial apoptosis through the PI3K/AKT signaling pathway.

Honokiol is a bioactive natural compound with a potential therapeutic benefit because of its diverse pharmacological properties, including antiinflammatory, anticancer, and antiarrhythmic, without appreciable toxicity [23–25]. This compound was also considered a potential treatment for heart disease [26]. Honokiol has been shown to enhance myocardial contraction and cardiac contractile capacity and reduce myocardial degeneration in the *β*1-AAB-induced myocardial dysfunction model [27]. Furthermore, it was also shown in previous studies that Honokiol decreases intima-to-media area ratio and intimal hyperplasia, as well as deposition of collagen in rabbit carotid artery balloon injury model [28]. Honokiol was also found to have antihypertrophic effects and may block cardiac fibroblast proliferation and differentiation to myofibroblasts [16]. Moreover, it was previously observed that Honokiol treatment provides cardiac protection from Dox-cardiotoxicity by enhancing mitochondrial function, implying that Honokiol is an auspicious treatment for cancer patients under Dox treatment [29]. Honokiol also performs a protective function against I/R in multiple organs, including the heart [15, 30–32]. Currently, there is only a little evidence about the effect of Honokiol on MI/RI. It was shown in a previous study that Honokiol ameliorates MI/RI in rats with type 1 diabetes by decreasing apoptosis and oxidative stress [14]. In addition, pretreatment with Honokiol was also observed to significantly reduce infarct size and the levels of proinflammatory cytokines (TNF-*α*, NF-*κ*B, and IL-6), oxidative stress indicators (myeloperoxidase, superoxide dismutase, catalase, malondialdehyde), and myocardial injury indicators (cTnT and CK-MB) in MI/RI [15].

The data from this research are in harmony with earlier studies. The results have shown that Honokiol pretreatment improves cardiac function during MI/RI and decreases the levels of myocardial injury indicators (cTnT and CK-MB) in the blood plasma. Moreover, Honokiol pretreatment led to the reduction of proapoptotic protein expression and mitochondrial swelling, both of which are induced by MI/RI.

Multiple signal pathways have been found to participate in the cardioprotective effects of Honokiol. Regulation of the NF-*κ*B pathway by Honokiol may reduce ROS levels and endothelial cell apoptosis in high-glucose-stimulated oxidative stress and streptozocin- (STZ-) stimulated diabetes [33], inflammatory response, apoptosis of human umbilical vein endothelial cells [34], and even the proliferation and migration of rat aortic smooth muscle cells [35]. Honokiol may be affecting cardiomyocytes autophagy via the AMPK/ULK pathway [36], as well as interstitial fibrosis and cardiac hypertrophy via the PI3K/AKT pathway [16]. As observed in previous studies, the NF-*κ*B pathway and the SIRT1-Nrf2 signaling pathway are implicated in the protective function of Honokiol in MI/RI [14, 15]. Our data suggest that the PI3K/AKT pathway is altered in the Honokiol pretreated cohort, suggesting its involvement in the cardioprotective effects of Honokiol in MI/RI. PI3K/AKT pathway triggering could reduce the levels of the proapoptotic protein Bax and stimulate the release of the antiapoptotic protein Bcl-2 [37]. Moreover, the caspase activation stimulates the release of Cyt-C and AIF, both of which facilitate the modulation of mitochondrial apoptosis [38–40]. Our results also showed that Honokiol pretreatment resulted in the decrease in the levels of Cyt-C, Bax, and cleaved caspase-9, as well as mitochondrial swelling in MI/RI, further suggesting that Honokiol may be protecting the heart from MI/RI by inhibiting mitochondrial apoptosis via the PI3K/AKT signaling pathway.

There are several potential limitations in this research that may be addressed in future related studies. First, the study is only aimed at investigating the protective effects of Honokiol in MI/RI without covering possible adverse effects. Next, the method of administration (intraperitoneal injection), as opposed to oral or rectal administration, may also influence the pharmacokinetics of the drug. After the Houpo extract was administered rectally to Wistar rats at a dosage of 245 mg/kg (equal to 13.5 mg/kg of Honokiol), the Honokiol's bioavailability was roughly sixfold greater than when orally administered at a similar dose [41]. This implies possible influences of the administration route in the therapeutic effects of Honokiol. For improved clinical applications, we will contrast the impacts of oral, rectal, and intraperitoneal injection in the bioavailability of Honokiol in MI/RI mouse models. Moreover, in order to further confirm the direct regulation of Honokiol on the PI3K/AKT pathway, the knock-out mice should be to use for verification.

## 5. Conclusion

In conclusion, Honokiol significantly decreases the levels of myocardial injury indicators (cTnT and CK-MB) induced by MI/RI. Honokiol inhibits cardiomyocyte apoptosis and mitochondrial swelling resulting from MI/RI and may involve the PI3K/AKT signaling pathway. Taken together, pretreatment with Honokiol provides cardiac protection from MI/RI by suppressing mitochondrial apoptosis through the PI3K/AKT signaling pathway.

## Data Availability

The authors will provide raw data to support the inferences of this research without reservations.
